# Racemic tricarbon­yl(η^6^-7-meth­oxy­flavan)chromium(0)

**DOI:** 10.1107/S1600536810024992

**Published:** 2010-07-10

**Authors:** Johannes. H. van Tonder, Barend C. B. Bezuidenhoudt, J. Marthinus Janse van Rensburg

**Affiliations:** a Department of Chemistry, University of the Free State, PO Box 339, Bloemfontein 9300, South Africa; b Organic Chemistry, Department of Chemisry, Lund University, PO Box 124, S-221 00, Lund, Sweden

## Abstract

In the title compound [systematic name: tricarbonyl(η^6^-7-methoxy-2-phenyl-3,4-dihydro-2*H*-1-benzopyran)chromium(0)], [Cr(C_16_H_16_O_2_)(CO)_3_], the Cr(CO)_3_ unit is coordinated by the phenyl­ene ring of the flavan ligand, exhibiting a three-legged piano-stool conformation, with a point to plane distance of 1.750 (1) Å. The phenyl ring is twisted away from the fused ring system by 36.49 (5)° (r.m.s. deviation = 0.027 Å; fitted atoms are the C_6_ ring and the attached fused-ring C and O atoms). The dihydro­pyran ring displays a distorted envelope configuration by displacement of the phenyl-bearing and the adjacent ring C atoms from the fused-ring system plane by 0.356 (2) and 0.402 (2) Å, respectively.

## Related literature

7-Meth­oxy­flavan was synthesized *via* hydrogenation from 7-meth­oxy­flavanone, as described by Sato *et al.* (2006 ▶). For coordination of 7-meth­oxy­flavan to chromium, see: Müller *et al.* (1999 ▶). For the importance of flavonoids in biological investigations, see: Rice-Evans & Packer (2003 ▶). For Cr(CO)_3_ coordination to the phenyl­ene ring of a flavanone compound, see: Dominique *et al.* (1999 ▶). For comparison bond distances, see: Allen *et al.* (1987 ▶). For related structures, see: van Tonder *et al.* (2009*a* ▶,*b* ▶). For the use of tricarbon­yl(arene)chromium complexes in regioselective organic synthesis, see: Muschalek *et al.* (2007 ▶).
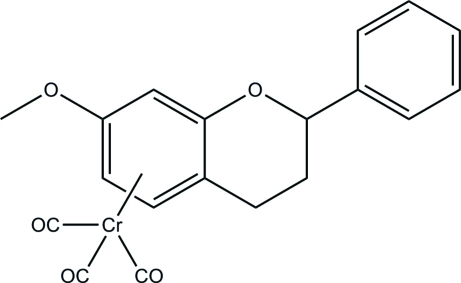


## Experimental

### 

#### Crystal data

[Cr(C_16_H_16_O_2_)(CO)_3_]
*M* *_r_* = 376.32
Monoclinic, 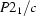

*a* = 9.8422 (2) Å
*b* = 12.3850 (3) Å
*c* = 15.0146 (3) Å
β = 115.171 (1)°
*V* = 1656.42 (6) Å^3^
*Z* = 4
Mo *K*α radiation
μ = 0.72 mm^−1^
*T* = 173 K
0.41 × 0.34 × 0.24 mm

#### Data collection

Bruker APEXII CCD diffractometer
Absorption correction: multi-scan (*SADABS*; Bruker, 2004 ▶) *T*_min_ = 0.757, *T*_max_ = 0.847
12982 measured reflections
3985 independent reflections
3224 reflections with *I* > 2σ(*I*)
*R*_int_ = 0.030

#### Refinement

*R*[*F* ^2^ > 2σ(*F* ^2^)] = 0.030
*wR*(*F* ^2^) = 0.085
*S* = 1.07
3985 reflections
227 parameters
H-atom parameters constrained
Δρ_max_ = 0.25 e Å^−3^
Δρ_min_ = −0.40 e Å^−3^

### 

Data collection: *APEX2* (Bruker, 2005 ▶); cell refinement: *SAINT-Plus* (Bruker, 2004 ▶); data reduction: *SAINT-Plus* and *XPREP* (Bruker, 2004 ▶); program(s) used to solve structure: *SIR97* (Altomare *et al.*, 1999 ▶); program(s) used to refine structure: *SHELXL97* (Sheldrick, 2008 ▶); molecular graphics: *DIAMOND* (Brandenburg & Putz, 2005 ▶); software used to prepare material for publication: *WinGX* (Farrugia, 1999 ▶).

## Supplementary Material

Crystal structure: contains datablocks global, I. DOI: 10.1107/S1600536810024992/zb2005sup1.cif

Structure factors: contains datablocks I. DOI: 10.1107/S1600536810024992/zb2005Isup2.hkl

Additional supplementary materials:  crystallographic information; 3D view; checkCIF report

## Figures and Tables

**Fig. 1.**
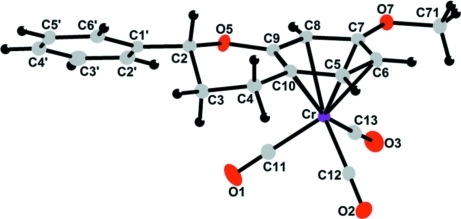
A view of (I) showing the atom-numbering scheme with displacement ellipsoids at the 30% probability level.

**Fig. 2.**
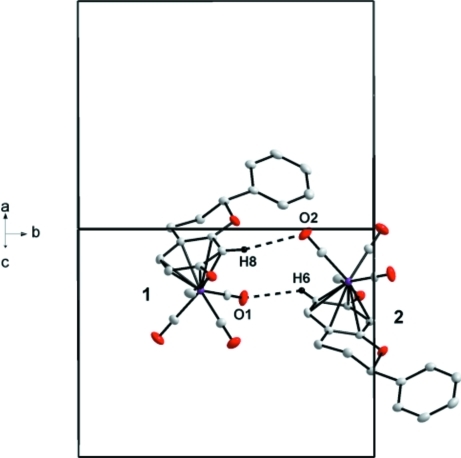
Indication of molecular packing in the unit-cell. Symmetry operators 1) x; y; z. 2) 1 - x; 0.5 + y; 1.5 - z.

## supplementary crystallographic information

### Comment

The title compound, (I), [Cr(C_16_H_16_O_2_)(CO)_3_], where (C_16_H_16_O_2_) = 7-methoxyflavan, has been examined due to the general biological activity of flavanoids (Rice-Evans & Packer, 2003) and the use of tricarbonyl(arene)chromium complexes in regioselective organic synthesis (Muschalek *et al.*, 2007).

As with the tricarbonyl(η^6^-flavanone)chromium(0) complex reported by Dominique *et al.* (1999), the Cr(CO)_3_ unit of the title compound is coordinated by the phenylene ring of the flavanoid backbone (Fig.1). The chromium metal centre is displaced by 1.750 (1) Å from the A-η^6^-coordinated arene ring centre. The dihydropyran ring displays a distorted envelope configuration by displacement of atoms C2 and C3 from the fused ring system plane, with distances of 0.356 (2) and 0.402 (2) Å respectively (r.m.s. of fitted atoms C4, C10, C5, C6, C7, C8, C9 and O5 = 0.027 °). Further molecular disorder is displayed by the phenyl ring twist away from the fused ring system plane, by 36.49 (5)°.

The molecular packing displays two types of soft intermolecular contacts, this between O2···H8 [2.682 (1) Å] forming a O2···H8—C8 angle of 152.5 (1)° and O1···H6 [2.459 (1) Å] forming a O1···H6—C6 angle of 125.9 (1)° (Fig.2).

### Experimental

7-Methoxyflavan was synthesised via H_2_SO_4_ catalyzed hydrogenation (5 bar) over 10% Pd/C from 7-methoxyflavanone, as described by Sato et al. (2006). 7-Methoxyflavan-4-one (1.00 g; 3.9 mmol), 10 % Pd/C (0.10 g), 3 M H_2_SO_4_ (aq.) (1 ml), EtOH (30 ml). Purification by means of flash column-chromatography yielded 7-methoxyflavan (0.67 g; 70.6%) as a colourless oil.

R_f_ 0.65 (H:DCM:EtOAc; 50:50:1); ^1^H NMR (600 MHz, CDCl_3_) δ ppm 7.44 – 7.41 (2H, m, H-2' and H-6'), 7.40 – 7.37 (2H, m, H-3' and H-5'), 7.34 – 7.31 (1H, m, H-4'), 6.99 – 6.97 (1H, m, H-5), 6.50 – 6.47 (2H, m, H-6 and H-8), 5.05 (1H, dd, J = 2.37, 10.19 Hz, H-2), 3.77 (3H, s, -OCH_3_), 2.92 (1H, ddd, J = 6.02, 10.92, 16.08 Hz, H-4(a)), 2.74 (1H, ddd, J = 3.40, 5.12, 16.08 Hz, H-4(e)), 2.22 – 2.18 (1H, m, H-3), 2.11– 2.04 (1H, m, H-3); ^13^C NMR (600 MHz, CDCl3) δ ppm 24.47 (C-4), 30.19 (C-3), 55.38 (-OCH_3_), 77.98 (C-2), 101.71 (C-6/8), 107.54 (C-6/8), 114.01, 126.11, 127.93, 128.61, 130.05, 141.79, 155.91, 155.91, 159.23

Preparation of the title compound, tricarbonyl(A-η^6^-7-methoxyflavane)chromium(0), was based on a method described by Müller et al. (1999). A solution of 7-Methoxyflavane (0.27 g, 1.1 mmol) and Cr(CO)_6_ (0.25 g, 1.1 mmol: 1 eq.) in Bu_2_O:THF (9:1; 10 ml per 100 mg Cr(CO)_6_ was degassed with argon, using standard Schlenk techniques, and refluxed (48 h) under an oxygen free atmosphere. The reaction mixture was cooled to room temperature and the solvent evaporated in vacuo. Purification through flash column-chromatography yielded tricarbonyl(A-η^6^-7-methoxyflavane)chromium(0) (0.07 g; 16.6.0%) as a yellow solid. Recrystallization from diethyl ether yielded yellow cuboidal crystals.

R_f_ 0.23 (Hexane: Acetone; 8:2); Mp 148.4 °C; Note: A, B and C-ring labelling refers to the benzene, phenyl and dihydropyrane rings respectively. ^1^H NMR (600 MHz, CDCl_3_) δ ppm 7.49 (2H, d, J = 7.15 Hz, H-2' and H-6'), 7.41 (2H, dd, J = 7.15, 8.66 Hz, H-3' and H-5'), 7.39 – 7.35 (1H, m, H-4'), 5.65 (1H, d, J = 6.61 Hz, H-5), 5.15 (1H, s, H-8), 4.90 – 4.86 (2H, m, H-2 and H-6), 3.72 (3H, s, -OCH_3_), 2.93 (1H, ddd, J = 4.89, 12.43, 15.65 Hz, H-4(a)), 2.54 (1H, dd, J = 4.14, 15.65 Hz, H-4(e)), 2.31 (1H, ddd, J = 4.14, 12.43, 13.68 Hz, H-3(a)), 2.13 (1H, dd, J = 4.89, 13.68 Hz, H-3(e)); ^13^C NMR (600 MHz, CDCl_3_) δ ppm 25.52 (C-4), 29.52 (C-3), 55.86 (-OCH_3_), 68.31 (C-8), 74.42 (C-2/6), 80.56 (C-2/6), 89.11, 94.55 (C-5), 126.61, 128.83, 128.89, 139.62, 140.30, 143.33, 234.44 (-Cr(CO)3); MS m/z 376 (M+, 13.0), 344 (0.2), 320 (0.1), 292 (70.7), 277 (0.2), 256 (0.1), 240 (5.8), 225 (0.5), 209 (0.3), 188 (100.0), 173 (0.4), 146 (10.0), 137 (2.1), 121 (2.0), 104 (5.1).

### Refinement

The H atoms were positioned geometrically and refined using a riding model with fixed C—H distances of 0.93 Å (ArH) [*U*_iso_(H) = 1.2*U*_eq_], 1.00 Å (CH) [*U*_iso_(H) = 1.2*U*_eq_], 0.99 Å (CH_2_) [*U*_iso_(H) = 1.2*U*_eq_] and 0.96 Å (CH_3_) [*U*_iso_(H) = 1.5*U*_eq_]. Initial positions of methyl H-atoms were obtained from fourier difference and refined as a fixed rotor.

The highest density peak is 0.25 located 0.66 Å from C1' and the deepest hole is -0.40 located at 0.50 Å from Cr.

### Crystal data

**Table:**

[Cr(C_16_H_16_O_2_)(CO)_3_]	*F*(000) = 776
*M**_r_* = 376.32	*D*_x_ = 1.509 Mg m^−^^3^
Monoclinic, *P*2_1_/*c*	Mo *K*α radiation, λ = 0.71073 Å
Hall symbol: -P 2ybc	Cell parameters from 5285 reflections
*a* = 9.8422 (2) Å	θ = 2.2–28.3°
*b* = 12.3850 (3) Å	µ = 0.72 mm^−^^1^
*c* = 15.0146 (3) Å	*T* = 173 K
β = 115.171 (1)°	Prism, yellow
*V* = 1656.42 (6) Å^3^	0.41 × 0.34 × 0.24 mm
*Z* = 4	

### Data collection

**Table:**

Bruker APEXII CCD diffractometer	3224 reflections with *I* > 2σ(*I*)
φ and ω scans	*R*_int_ = 0.030
Absorption correction: multi-scan (Bruker, 2004)	θ_max_ = 28°, θ_min_ = 2.2°
*T*_min_ = 0.757, *T*_max_ = 0.847	*h* = −11→12
12982 measured reflections	*k* = −16→16
3985 independent reflections	*l* = −19→19

### Refinement

**Table:**

Refinement on *F*^2^	0 restraints
Least-squares matrix: full	H-atom parameters constrained
*R*[*F*^2^ > 2σ(*F*^2^)] = 0.030	*w* = 1/[σ^2^(*F*_o_^2^) + (0.0444*P*)^2^ + 0.0884*P*] where *P* = (*F*_o_^2^ + 2*F*_c_^2^)/3
*wR*(*F*^2^) = 0.085	(Δ/σ)_max_ = 0.001
*S* = 1.07	Δρ_max_ = 0.25 e Å^−^^3^
3985 reflections	Δρ_min_ = −0.40 e Å^−^^3^
227 parameters	

### Special details

**Table:**

Experimental. The intensity data was collected on a Bruker Apex II CCD diffractometer using a frame width of 0.5° covering up to θ = 28° with 100 % completeness accomplished.
Geometry. All esds (except the esd in the dihedral angle between two l.s. planes) are estimated using the full covariance matrix. The cell esds are taken into account individually in the estimation of esds in distances, angles and torsion angles; correlations between esds in cell parameters are only used when they are defined by crystal symmetry. An approximate (isotropic) treatment of cell esds is used for estimating esds involving l.s. planes.

### Fractional atomic coordinates and isotropic or equivalent isotropic displacement parameters (Å^2^)

**Table:**

	*x*	*y*	*z*	*U*_iso_*/*U*_eq_	
C1'	1.01976 (18)	0.59116 (14)	0.84890 (12)	0.0260 (3)	
C2'	0.9566 (2)	0.69062 (15)	0.85055 (13)	0.0325 (4)	
H2'	0.8506	0.6988	0.8213	0.039*	
C2	0.92726 (18)	0.49179 (14)	0.80339 (12)	0.0268 (4)	
H2	0.9703	0.4553	0.7618	0.032*	
C3'	1.0487 (2)	0.77884 (16)	0.89526 (14)	0.0385 (4)	
H3'	1.0052	0.8471	0.8958	0.046*	
C3	0.92236 (18)	0.41073 (13)	0.87814 (12)	0.0276 (4)	
H3A	0.8755	0.4443	0.9181	0.033*	
H3B	1.0257	0.3886	0.923	0.033*	
C4	0.83175 (18)	0.31200 (14)	0.82448 (12)	0.0272 (4)	
H4A	0.8908	0.2687	0.798	0.033*	
H4B	0.8107	0.2663	0.8713	0.033*	
C4'	1.2029 (2)	0.76723 (16)	0.93867 (13)	0.0380 (4)	
H4'	1.2654	0.8272	0.9696	0.046*	
C5'	1.2657 (2)	0.66846 (16)	0.93694 (13)	0.0360 (4)	
H5'	1.3717	0.6603	0.967	0.043*	
C5	0.56441 (18)	0.27492 (13)	0.69612 (12)	0.0260 (4)	
H5	0.5723	0.2037	0.7214	0.031*	
C6	0.43161 (18)	0.30503 (13)	0.61485 (12)	0.0263 (3)	
H6	0.3532	0.2541	0.5842	0.032*	
C6'	1.17553 (18)	0.58135 (15)	0.89185 (12)	0.0295 (4)	
H6'	1.2201	0.5138	0.89	0.035*	
C7	0.41667 (18)	0.41122 (13)	0.57975 (11)	0.0247 (3)	
C8	0.53223 (18)	0.48677 (13)	0.62755 (12)	0.0251 (3)	
H8	0.5183	0.5606	0.6085	0.03*	
C9	0.66817 (17)	0.45213 (14)	0.70352 (12)	0.0242 (3)	
C10	0.68615 (17)	0.34612 (13)	0.74146 (12)	0.0237 (3)	
C11	0.56076 (19)	0.50205 (13)	0.85013 (13)	0.0269 (4)	
C12	0.42375 (19)	0.32289 (14)	0.82151 (13)	0.0295 (4)	
C13	0.29284 (19)	0.48252 (14)	0.70530 (13)	0.0298 (4)	
C71	0.17072 (19)	0.37892 (16)	0.45371 (13)	0.0335 (4)	
H71A	0.2029	0.3181	0.4255	0.05*	
H71B	0.0894	0.4179	0.401	0.05*	
H71C	0.1349	0.3518	0.5013	0.05*	
O1	0.61634 (16)	0.55726 (10)	0.91861 (10)	0.0436 (4)	
O2	0.39278 (16)	0.26735 (12)	0.87217 (10)	0.0486 (4)	
O3	0.17872 (15)	0.52563 (12)	0.67906 (11)	0.0482 (4)	
O5	0.77684 (12)	0.52853 (9)	0.73998 (9)	0.0305 (3)	
O7	0.29490 (12)	0.45045 (10)	0.50235 (8)	0.0303 (3)	
Cr	0.47389 (3)	0.413031 (19)	0.743218 (18)	0.01990 (9)	

### Atomic displacement parameters (Å^2^)

**Table:**

	*U*^11^	*U*^22^	*U*^33^	*U*^12^	*U*^13^	*U*^23^
C1'	0.0229 (8)	0.0363 (9)	0.0193 (8)	−0.0040 (7)	0.0096 (7)	0.0017 (7)
C2'	0.0282 (9)	0.0401 (10)	0.0317 (10)	−0.0012 (8)	0.0152 (8)	0.0017 (8)
C2	0.0198 (8)	0.0364 (9)	0.0228 (8)	−0.0006 (7)	0.0077 (7)	0.0013 (7)
C3'	0.0494 (11)	0.0344 (10)	0.0391 (11)	−0.0015 (9)	0.0259 (10)	0.0000 (8)
C3	0.0239 (8)	0.0319 (9)	0.0230 (8)	0.0030 (7)	0.0062 (7)	0.0046 (7)
C4	0.0241 (8)	0.0281 (8)	0.0265 (9)	0.0044 (7)	0.0079 (7)	0.0039 (7)
C4'	0.0444 (11)	0.0425 (11)	0.0286 (10)	−0.0176 (9)	0.0170 (9)	−0.0023 (8)
C5'	0.0278 (9)	0.0524 (12)	0.0263 (9)	−0.0110 (9)	0.0100 (8)	0.0034 (8)
C5	0.0286 (8)	0.0203 (8)	0.0280 (9)	0.0025 (7)	0.0110 (7)	−0.0027 (6)
C6	0.0261 (8)	0.0254 (8)	0.0256 (8)	−0.0011 (7)	0.0092 (7)	−0.0063 (7)
C6'	0.0248 (8)	0.0389 (10)	0.0247 (9)	−0.0013 (7)	0.0105 (7)	0.0037 (7)
C7	0.0209 (8)	0.0331 (9)	0.0195 (8)	0.0020 (7)	0.0081 (6)	0.0009 (7)
C8	0.0243 (8)	0.0279 (8)	0.0232 (8)	−0.0002 (7)	0.0102 (7)	0.0061 (7)
C9	0.0211 (8)	0.0306 (8)	0.0216 (8)	−0.0023 (7)	0.0098 (7)	0.0017 (7)
C10	0.0229 (8)	0.0258 (8)	0.0227 (8)	0.0021 (6)	0.0101 (7)	−0.0003 (6)
C11	0.0281 (9)	0.0203 (8)	0.0292 (9)	0.0040 (7)	0.0090 (7)	0.0041 (7)
C12	0.0291 (9)	0.0308 (9)	0.0269 (9)	−0.0074 (7)	0.0104 (7)	−0.0030 (7)
C13	0.0274 (9)	0.0331 (9)	0.0280 (9)	−0.0003 (7)	0.0108 (7)	−0.0054 (7)
C71	0.0206 (8)	0.0466 (11)	0.0286 (9)	−0.0041 (8)	0.0058 (7)	0.0003 (8)
O1	0.0546 (9)	0.0264 (6)	0.0327 (7)	0.0063 (6)	0.0020 (7)	−0.0075 (6)
O2	0.0537 (9)	0.0537 (9)	0.0381 (8)	−0.0232 (7)	0.0195 (7)	0.0062 (7)
O3	0.0302 (7)	0.0592 (9)	0.0495 (9)	0.0148 (7)	0.0115 (7)	−0.0084 (7)
O5	0.0203 (6)	0.0333 (7)	0.0295 (6)	−0.0058 (5)	0.0024 (5)	0.0110 (5)
O7	0.0231 (6)	0.0369 (7)	0.0243 (6)	−0.0018 (5)	0.0037 (5)	0.0050 (5)
Cr	0.02079 (14)	0.01811 (14)	0.02113 (14)	−0.00032 (10)	0.00923 (11)	0.00022 (10)

### Geometric parameters (Å, °)

**Table:**

C1'—C2'	1.385 (3)	C6—C7	1.401 (2)
C1'—C6'	1.393 (2)	C6—Cr	2.2350 (16)
C1'—C2	1.510 (2)	C6—H6	0.95
C2'—C3'	1.396 (3)	C6'—H6'	0.95
C2'—H2'	0.95	C7—O7	1.3558 (19)
C2—O5	1.4513 (19)	C7—C8	1.411 (2)
C2—C3	1.522 (2)	C7—Cr	2.2729 (16)
C2—H2	1	C8—C9	1.406 (2)
C3'—C4'	1.381 (3)	C8—Cr	2.2424 (16)
C3'—H3'	0.95	C8—H8	0.95
C3—C4	1.526 (2)	C9—O5	1.3568 (19)
C3—H3A	0.99	C9—C10	1.412 (2)
C3—H3B	0.99	C9—Cr	2.2838 (16)
C4—C10	1.505 (2)	C10—Cr	2.2580 (16)
C4—H4A	0.99	C11—O1	1.160 (2)
C4—H4B	0.99	C11—Cr	1.8319 (17)
C4'—C5'	1.375 (3)	C12—O2	1.159 (2)
C4'—H4'	0.95	C12—Cr	1.8344 (18)
C5'—C6'	1.378 (2)	C13—O3	1.151 (2)
C5'—H5'	0.95	C13—Cr	1.8376 (18)
C5—C6	1.407 (2)	C71—O7	1.433 (2)
C5—C10	1.408 (2)	C71—H71A	0.98
C5—Cr	2.1808 (16)	C71—H71B	0.98
C5—H5	0.95	C71—H71C	0.98
			
C2'—C1'—C6'	118.91 (16)	O5—C9—C8	115.42 (14)
C2'—C1'—C2	122.96 (15)	O5—C9—C10	122.93 (14)
C6'—C1'—C2	118.12 (15)	C8—C9—C10	121.58 (15)
C1'—C2'—C3'	120.00 (16)	O5—C9—Cr	130.27 (11)
C1'—C2'—H2'	120	C8—C9—Cr	70.31 (9)
C3'—C2'—H2'	120	C10—C9—Cr	70.90 (9)
O5—C2—C1'	106.96 (13)	C5—C10—C9	116.89 (14)
O5—C2—C3	110.32 (13)	C5—C10—C4	122.62 (14)
C1'—C2—C3	113.95 (14)	C9—C10—C4	120.46 (14)
O5—C2—H2	108.5	C5—C10—Cr	68.55 (9)
C1'—C2—H2	108.5	C9—C10—Cr	72.88 (9)
C3—C2—H2	108.5	C4—C10—Cr	130.77 (11)
C4'—C3'—C2'	120.27 (18)	O1—C11—Cr	179.12 (15)
C4'—C3'—H3'	119.9	O2—C12—Cr	178.82 (16)
C2'—C3'—H3'	119.9	O3—C13—Cr	178.24 (16)
C2—C3—C4	109.51 (14)	O7—C71—H71A	109.5
C2—C3—H3A	109.8	O7—C71—H71B	109.5
C4—C3—H3A	109.8	H71A—C71—H71B	109.5
C2—C3—H3B	109.8	O7—C71—H71C	109.5
C4—C3—H3B	109.8	H71A—C71—H71C	109.5
H3A—C3—H3B	108.2	H71B—C71—H71C	109.5
C10—C4—C3	110.43 (13)	C9—O5—C2	117.07 (12)
C10—C4—H4A	109.6	C7—O7—C71	117.83 (13)
C3—C4—H4A	109.6	C11—Cr—C12	87.55 (7)
C10—C4—H4B	109.6	C11—Cr—C13	91.11 (7)
C3—C4—H4B	109.6	C12—Cr—C13	89.65 (8)
H4A—C4—H4B	108.1	C11—Cr—C5	130.80 (7)
C5'—C4'—C3'	119.72 (18)	C12—Cr—C5	89.36 (7)
C5'—C4'—H4'	120.1	C13—Cr—C5	137.98 (7)
C3'—C4'—H4'	120.1	C11—Cr—C6	164.20 (7)
C4'—C5'—C6'	120.35 (17)	C12—Cr—C6	100.73 (7)
C4'—C5'—H5'	119.8	C13—Cr—C6	102.28 (7)
C6'—C5'—H5'	119.8	C5—Cr—C6	37.14 (6)
C6—C5—C10	122.61 (15)	C11—Cr—C8	104.82 (7)
C6—C5—Cr	73.52 (9)	C12—Cr—C8	166.28 (7)
C10—C5—Cr	74.51 (9)	C13—Cr—C8	95.93 (7)
C6—C5—H5	118.7	C5—Cr—C8	78.10 (6)
C10—C5—H5	118.7	C6—Cr—C8	65.85 (6)
Cr—C5—H5	125	C11—Cr—C10	98.15 (7)
C7—C6—C5	119.02 (15)	C12—Cr—C10	106.65 (7)
C7—C6—Cr	73.38 (9)	C13—Cr—C10	161.49 (7)
C5—C6—Cr	69.34 (9)	C5—Cr—C10	36.94 (6)
C7—C6—H6	120.5	C6—Cr—C10	66.68 (6)
C5—C6—H6	120.5	C8—Cr—C10	66.26 (6)
Cr—C6—H6	129	C11—Cr—C7	139.56 (7)
C5'—C6'—C1'	120.73 (17)	C12—Cr—C7	132.48 (7)
C5'—C6'—H6'	119.6	C13—Cr—C7	84.71 (7)
C1'—C6'—H6'	119.6	C5—Cr—C7	65.78 (6)
O7—C7—C6	124.88 (15)	C6—Cr—C7	36.20 (6)
O7—C7—C8	115.21 (14)	C8—Cr—C7	36.40 (6)
C6—C7—C8	119.89 (14)	C10—Cr—C7	77.84 (6)
O7—C7—Cr	130.14 (11)	C11—Cr—C9	88.31 (7)
C6—C7—Cr	70.42 (9)	C12—Cr—C9	141.22 (7)
C8—C7—Cr	70.62 (9)	C13—Cr—C9	128.98 (7)
C9—C8—C7	119.56 (15)	C5—Cr—C9	65.09 (6)
C9—C8—Cr	73.51 (9)	C6—Cr—C9	76.78 (6)
C7—C8—Cr	72.98 (9)	C8—Cr—C9	36.18 (6)
C9—C8—H8	120.2	C10—Cr—C9	36.22 (6)
C7—C8—H8	120.2	C7—Cr—C9	64.57 (6)
Cr—C8—H8	124.9		
			
C6'—C1'—C2'—C3'	−0.4 (3)	C7—C6—Cr—C9	−64.80 (10)
C2—C1'—C2'—C3'	178.97 (16)	C5—C6—Cr—C9	66.02 (10)
C2'—C1'—C2—O5	17.3 (2)	C9—C8—Cr—C11	65.59 (11)
C6'—C1'—C2—O5	−163.39 (14)	C7—C8—Cr—C11	−165.45 (10)
C2'—C1'—C2—C3	−104.93 (18)	C9—C8—Cr—C12	−88.2 (3)
C6'—C1'—C2—C3	74.42 (19)	C7—C8—Cr—C12	40.8 (3)
C1'—C2'—C3'—C4'	−0.5 (3)	C9—C8—Cr—C13	158.32 (10)
O5—C2—C3—C4	61.99 (17)	C7—C8—Cr—C13	−72.72 (10)
C1'—C2—C3—C4	−177.69 (14)	C9—C8—Cr—C5	−63.83 (10)
C2—C3—C4—C10	−47.68 (18)	C7—C8—Cr—C5	65.13 (10)
C2'—C3'—C4'—C5'	0.6 (3)	C9—C8—Cr—C6	−100.86 (11)
C3'—C4'—C5'—C6'	0.3 (3)	C7—C8—Cr—C6	28.10 (9)
C10—C5—C6—C7	−2.7 (2)	C9—C8—Cr—C10	−26.92 (10)
Cr—C5—C6—C7	56.02 (14)	C7—C8—Cr—C10	102.04 (10)
C10—C5—C6—Cr	−58.68 (14)	C9—C8—Cr—C7	−128.96 (15)
C4'—C5'—C6'—C1'	−1.2 (3)	C7—C8—Cr—C9	128.96 (15)
C2'—C1'—C6'—C5'	1.2 (3)	C5—C10—Cr—C11	155.21 (10)
C2—C1'—C6'—C5'	−178.16 (16)	C9—C10—Cr—C11	−75.77 (10)
C5—C6—C7—O7	−179.96 (15)	C4—C10—Cr—C11	40.00 (16)
Cr—C6—C7—O7	−125.88 (16)	C5—C10—Cr—C12	65.34 (11)
C5—C6—C7—C8	−1.7 (2)	C9—C10—Cr—C12	−165.64 (10)
Cr—C6—C7—C8	52.36 (14)	C4—C10—Cr—C12	−49.87 (16)
C5—C6—C7—Cr	−54.07 (13)	C5—C10—Cr—C13	−85.5 (2)
O7—C7—C8—C9	−174.85 (14)	C9—C10—Cr—C13	43.5 (3)
C6—C7—C8—C9	6.7 (2)	C4—C10—Cr—C13	159.3 (2)
Cr—C7—C8—C9	59.01 (14)	C9—C10—Cr—C5	129.02 (14)
O7—C7—C8—Cr	126.14 (13)	C4—C10—Cr—C5	−115.21 (18)
C6—C7—C8—Cr	−52.27 (14)	C5—C10—Cr—C6	−29.40 (9)
C7—C8—C9—O5	175.10 (14)	C9—C10—Cr—C6	99.61 (10)
Cr—C8—C9—O5	−126.16 (14)	C4—C10—Cr—C6	−144.62 (16)
C7—C8—C9—C10	−7.7 (2)	C5—C10—Cr—C8	−102.12 (10)
Cr—C8—C9—C10	51.09 (14)	C9—C10—Cr—C8	26.90 (9)
C7—C8—C9—Cr	−58.74 (14)	C4—C10—Cr—C8	142.67 (16)
C6—C5—C10—C9	1.9 (2)	C5—C10—Cr—C7	−65.70 (10)
Cr—C5—C10—C9	−56.37 (13)	C9—C10—Cr—C7	63.32 (10)
C6—C5—C10—C4	−176.23 (15)	C4—C10—Cr—C7	179.09 (16)
Cr—C5—C10—C4	125.55 (15)	C5—C10—Cr—C9	−129.02 (14)
C6—C5—C10—Cr	58.22 (14)	C4—C10—Cr—C9	115.77 (18)
O5—C9—C10—C5	−179.62 (15)	O7—C7—Cr—C11	−85.11 (17)
C8—C9—C10—C5	3.3 (2)	C6—C7—Cr—C11	155.29 (11)
Cr—C9—C10—C5	54.18 (13)	C8—C7—Cr—C11	21.99 (14)
O5—C9—C10—C4	−1.5 (2)	O7—C7—Cr—C12	85.04 (17)
C8—C9—C10—C4	−178.53 (15)	C6—C7—Cr—C12	−34.57 (13)
Cr—C9—C10—C4	−127.70 (15)	C8—C7—Cr—C12	−167.87 (11)
O5—C9—C10—Cr	126.20 (16)	O7—C7—Cr—C13	0.38 (15)
C8—C9—C10—Cr	−50.83 (14)	C6—C7—Cr—C13	−119.22 (11)
C3—C4—C10—C5	−163.05 (15)	C8—C7—Cr—C13	107.48 (11)
C3—C4—C10—C9	18.9 (2)	O7—C7—Cr—C5	149.67 (16)
C3—C4—C10—Cr	−74.28 (18)	C6—C7—Cr—C5	30.06 (9)
C8—C9—O5—C2	−167.67 (14)	C8—C7—Cr—C5	−103.24 (10)
C10—C9—O5—C2	15.1 (2)	O7—C7—Cr—C6	119.60 (19)
Cr—C9—O5—C2	107.28 (16)	C8—C7—Cr—C6	−133.30 (14)
C1'—C2—O5—C9	−169.74 (13)	O7—C7—Cr—C8	−107.10 (18)
C3—C2—O5—C9	−45.31 (19)	C6—C7—Cr—C8	133.30 (14)
C6—C7—O7—C71	1.2 (2)	O7—C7—Cr—C10	−173.42 (16)
C8—C7—O7—C71	−177.07 (14)	C6—C7—Cr—C10	66.97 (10)
Cr—C7—O7—C71	−91.82 (17)	C8—C7—Cr—C10	−66.33 (10)
C6—C5—Cr—C11	−164.93 (10)	O7—C7—Cr—C9	−137.65 (16)
C10—C5—Cr—C11	−33.24 (13)	C6—C7—Cr—C9	102.75 (10)
C6—C5—Cr—C12	108.86 (11)	C8—C7—Cr—C9	−30.55 (9)
C10—C5—Cr—C12	−119.45 (10)	O5—C9—Cr—C11	−11.15 (14)
C6—C5—Cr—C13	20.10 (15)	C8—C9—Cr—C11	−118.27 (11)
C10—C5—Cr—C13	151.78 (11)	C10—C9—Cr—C11	106.27 (10)
C10—C5—Cr—C6	131.69 (15)	O5—C9—Cr—C12	−95.13 (17)
C6—C5—Cr—C8	−65.53 (10)	C8—C9—Cr—C12	157.75 (12)
C10—C5—Cr—C8	66.16 (10)	C10—C9—Cr—C12	22.30 (15)
C6—C5—Cr—C10	−131.69 (15)	O5—C9—Cr—C13	78.91 (16)
C6—C5—Cr—C7	−29.35 (10)	C8—C9—Cr—C13	−28.21 (13)
C10—C5—Cr—C7	102.34 (10)	C10—C9—Cr—C13	−163.67 (10)
C6—C5—Cr—C9	−101.28 (11)	O5—C9—Cr—C5	−148.41 (16)
C10—C5—Cr—C9	30.41 (9)	C8—C9—Cr—C5	104.47 (11)
C7—C6—Cr—C11	−84.6 (3)	C10—C9—Cr—C5	−30.99 (9)
C5—C6—Cr—C11	46.3 (3)	O5—C9—Cr—C6	174.13 (15)
C7—C6—Cr—C12	154.79 (10)	C8—C9—Cr—C6	67.01 (10)
C5—C6—Cr—C12	−74.38 (11)	C10—C9—Cr—C6	−68.45 (10)
C7—C6—Cr—C13	62.79 (11)	O5—C9—Cr—C8	107.12 (18)
C5—C6—Cr—C13	−166.38 (11)	C10—C9—Cr—C8	−135.45 (15)
C7—C6—Cr—C5	−130.82 (15)	O5—C9—Cr—C10	−117.42 (18)
C7—C6—Cr—C8	−28.25 (9)	C8—C9—Cr—C10	135.45 (15)
C5—C6—Cr—C8	102.57 (11)	O5—C9—Cr—C7	137.85 (16)
C7—C6—Cr—C10	−101.56 (10)	C8—C9—Cr—C7	30.73 (10)
C5—C6—Cr—C10	29.26 (9)	C10—C9—Cr—C7	−104.73 (10)
C5—C6—Cr—C7	130.82 (15)		
